# Directing recreation pressure via pathways allows for coexistence of recreation and nature development on the upper beach

**DOI:** 10.1007/s10980-025-02246-2

**Published:** 2025-11-20

**Authors:** Sasja J. van Rosmalen, Jan-Markus Homberger, Michel Riksen, Juul Limpens

**Affiliations:** 1 Plant Ecology and Nature Conservation Group, Wageningen University & Research, Wageningen, Netherlands; 2 Soil Physics and Land Management Group, Wageningen University & Research, Wageningen, Netherlands

**Keywords:** Dune development, Recreation, Anthropogenic impacts, Plant establishment, Biodiversity

## Abstract

**Context:**

Sandy shorelines, including beaches and embryo dunes, are important spaces for both recreation and nature. Balancing these landscape functions remains a challenge. Directing recreation pressure via visitor footpaths (pathways) is widely used to mitigate recreation pressure in nature reserves. However, its potential to support multifunctionality on beaches is poorly quantified.

**Objectives:**

To assess the response relationship between directed recreation pressure and the establishment of characteristic plant species alongside associated topographical development on the upper beach.

**Methods:**

We experimentally introduced seeds and rhizomes (diaspores) of five common dune-building plant species in 30 locations at varying distance from main pathways at a beach near The Hague, the Netherlands. Species were a mix of grasses and herbs and ranged from pioneer to early successional species. We measured shoot emergence per species and initiation of embryo dunes, as well as environmental conditions and visitor numbers across one growing season.

**Results:**

We found that the likelihood of establishment and shoot density were significantly higher beyond the first 20 m from pathways, irrespective of species or diaspore type, suggesting a limited zone of influence of beach visitors. Similarly, the number of surviving species and embryonic dune initiations were significantly higher beyond the pathways. Indeed, visitor observations showed that most visitors followed existing pathways across the beach, confirming the limited zone of influence of recreation pressure in the presences of pathways.

**Conclusions:**

Recreation hampers the establishment of plant species and associated dune initiation on the upper beach. However, constraining recreation pressure on the upper beach by stimulating development of pathways can improve coexistence between nature development and recreation in coastal areas.

**Supplementary Information:**

The online version contains supplementary material available at 10.1007/s10980-025-02246-2.

## Introduction

The majority of the world’s population lives at or near the coast (Small and Nicholls 2003; Lansu et al. 2024). Sandy coasts account for a third of the world’s coastline (Luijendijk et al. 2018) and offer invaluable ecosystem services to humans. These services include flood protection, provision of drinking water, and space for recreational activities (Everard et al. 2010). Moreover, the complex gradients in environmental conditions in these systems provide habitat space for flora and fauna (Maun 2009), including species unique to embryo dunes and beaches. Balancing these ecosystem services and landscape functions in a limited space is a challenge for coastal management. Embryo dunes and beaches are most valued for their recreational use and their natural value. Yet, their natural value can be negatively impacted by recreation, when too many visitors damage the development of vegetation and associated topography (van Rosmalen et al. 2025). On the other hand, closing the area for visitors would improve natural values, it could harm local economies for which recreational activities are an important source of income (Bakhshianlamouki et al. 2023; Lansu et al. 2024). Finding a balance between enabling recreation and conserving natural values is especially important considering climate change. For example, with increasing temperatures coastal visitor numbers are expected to increase at the North Sea coasts (Coombes and Jones 2010), thereby amplifying anthropogenic pressure. Furthermore, climate predictions indicate sea level rise and increased storm frequency and intensity (Oppenheimer et al. 2019), which also can have negative impacts on vegetation development (Nolet and Riksen 2019; van Puijenbroek et al. 2017a, b).

Embryo dunes and sandy beaches host uniquely adapted vegetation, where the spatial distribution and niche of vegetation is determined by geomorphological drivers such as beach width and elevation and associated environmental drivers such as sediment dynamics, wave action, inundation, salinity, and soil moisture (Maun 2009; van Puijenbroek et al. 2017a, b; Nolet et al. 2018; Nolet and Riksen 2019; Homberger et al. 2025). These environmental drivers determine the presence and absence, as well as the abundance of species. On beaches with a recreational function, trampling by beach visitors is an additional determinant of plant establishment success (Šilc et al. 2017; Prisco et al. 2021). On the upper beach the negative impacts of trampling on the establishment of dune-building species can be found up to 120 meters longshore from beach entrance points (van Rosmalen et al. 2025). In this zone of influence newly germinated plants do not survive summer recreation. Given that dune building depends on the ability of plant species to capture and grow with the sediment, failures in plant establishment slow down or even arrest topography development. Consequently, trampling may affect the biodiversity value of the upper beach directly by killing seedlings, or indirectly by preventing the development of topography and its associated gradients in environmental drivers.

A common method of reducing the impact of trampling on vegetation is by localising the trampling pressure to a limited area, for example by directing people over pathways (Wolf et al. 2019). In the foredune and more landwards dunes this is mostly done by means of fencing or boardwalks and has proven to be an effective measure to minimize trampling impacts in surrounding areas (Hylgaard 1980; Andersen 1995; Kutiel et al. 1999; Kelly 2014). Likewise, on the upper beach an experiment with boardwalks showed to locally increase biodiversity by finding higher species numbers and increased vegetation cover in the areas with boardwalks compared to control plots (Prisco et al. 2021). Given the high cost associated with building boardwalks and keeping them free of sand, this is not a solution that can be applied everywhere. Instead of hard boundaries imposed by fences or boardwalks, pathways can also be delineated by soft boundaries such as the presence of adult vegetation or topography of the terrain itself. It is, however, unknown if pathways delineated by adult dune vegetation can serve the same role in localising recreation pressure as pathways delineated by other methods due to the patchy nature of dune vegetation.

In this study we investigated the relationship between recreation pressure and the establishment of five characteristic plant species by introducing their seeds and rhizomes at different distances from the main visitor footpaths (pathways) with soft boundaries on the upper beach and monitoring vegetation establishment success, and embryo dune initiation. We hypothesized that: (1) pathways with soft boundaries significantly constrain the impact of trampling, enabling plant species to establish successfully close to the pathways. As a result, (2) establishment success, growth and topographic development increase with distance to the pathway, and (3) the impact of trampling differs between species, with dune-building grasses (*Ammophila* *arenaria,* *Elytrigia* *juncea,* and *Leymus* *arenarius*) being less sensitive than herbs (*Honckenya* *peploides* and *Cakile* *maritima*).

## Material and methods

### Research area

**Fig. 1:**
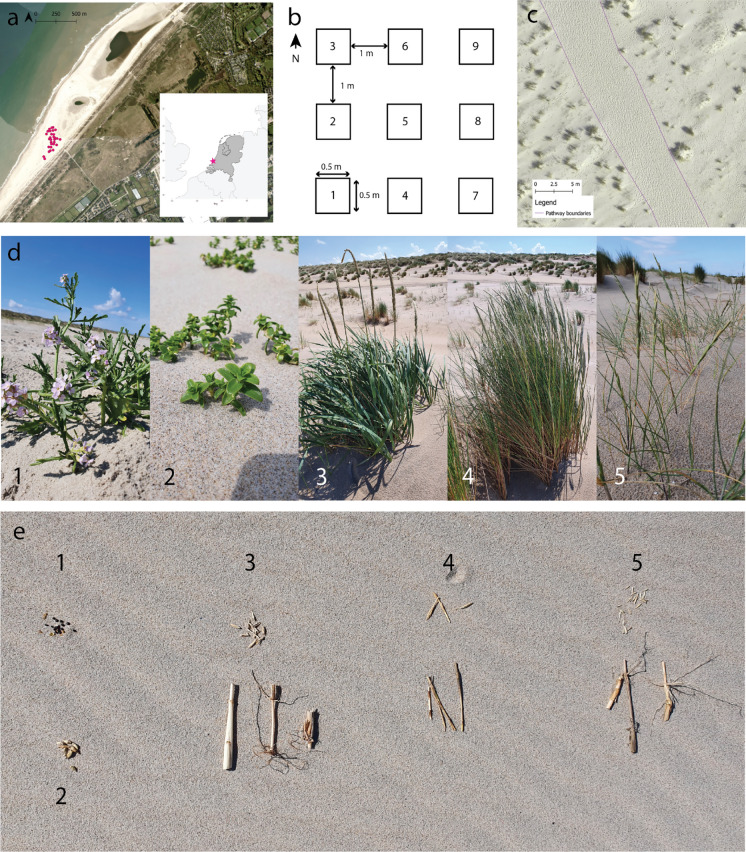
**a** Location of the research area on the Sand Engine at the Schelpenpad entrance where the dots indicate the block locations, insert: position in the Netherlands. **b** Standard plot lay out. **c** Close up of pathway boundary delineation. **d** Photos of the adult vegetation and E) Photos of their diaspores. (1) *Cakile* *maritima* seeds, (2) *Honckenya* *peploides* seeds, (3) *Leymus* *arenarius* seeds and rhizomes, (4) *Elytrigia* *juncea* seeds and rhizomes, (5) *Ammophila* *arenaria* seeds and rhizomes

For the experiment we selected a beach section on the Sand Engine, a mega-nourishment created between The Hague and the port of Rotterdam in the Netherlands in 2011 (Goossen et al. 2020). The beach section at the Schelpenpad entrance (lat: 52.04452, long: 4.18256, Fig 1a) was selected for three reasons: (1) it has an (naturally developed) existing pathway structure on the upper beach, (2) it supports vegetation and embryo dune development as shown by ample presence of both, and (3) no mechanical beach cleaning takes place at this beach section, permitting vegetation development. The Schelpenpad section had a beach width of approximately 350 m and an average beach height of 4.5 m (3.4–5.1 m), meeting the requirements for vegetation establishment (Nolet and Riksen 2019). The research area is part of the aeolian active zone of the Sand Engine (Hoonhout and de Vries 2017; Nolet and Riksen 2019) and is overall accreting sediment (Nolet and Riksen 2019; Van Westen et al. 2023).The sediment of the Sand Engine is coarser, and contains more shells, than sediments of the surrounding coast due to its origin of nourishment sediment from the Noth Sea floor (Hoonhout and de Vries 2017). The beach section is accessible via a beach entrance with bicycle parking, additionally it can also be reached by public transport and roadside parking. In contrast to other beach sections, there are no facilities such as restaurants available. As a result, this beach section, though well visited, sees fewer visitors compared to the more popular sections to the north and south. The experimental year of 2022 was sunny and consisted of a mild dry spring, a dominantly hot dry summer, and mild and rather wet autumn (Huiskamp 2023). Compared to the long-term average annual rainfall and temperature of the years 2001-2024, the experimental year is a little drier 746 vs 887 mm) and warmer (12 vs 11 °C). For detailed rainfall, temperature, and wind conditions during the experimental period see Online Appendix Fig 6 and 7.

### Experimental design

To test the impact of recreation on vegetation establishment, biodiversity, and embryo dune development an introduction experiment was set up in April 2022. At the Schelpenpad beach section, 30 locations were selected at different distances to the main pathways. The locations were selected using doubly balanced sampling with equal inclusion probabilities to ensure spatially representative yet random placement across the area. This approach was implemented using the R package “BalancedSampling” (Grafström and Tillé 2013; Grafström et al. 2024). We used covariates obtained from Digital Terrain Models (DTM) from Rijkswaterstaat, the executive agency of the Ministry of Infrastructure and Water Management. The models are available under a Creative Commons Zero (CC0) license (last access: 22.11.2023). As spreading variables, we used the centre coordinates of the raster pixels (x and y). The covariates, chosen for their documented importance in the establishment of beach vegetation, are the height above sea level (2021), average yearly bed level change (2016–2021), the topographical wetness index, and the distance to the beach entrance. The average yearly bed level change was calculated as follows (Eq 1):

1 $${\text{DTM}}_{{\Delta {\text{height}}}} = \, \left( {{\text{DTM}}_{{{2}0{17}}} - {\text{ DTM}}_{{{2}0{16}}} +_{{}} {\text{DTM}}_{{{2}0{18}}} - {\text{ DTM}}_{{{2}0{17}}} +_{{}} {\text{DTM}}_{{{2}0{19}}} - {\text{ DTM}}_{{{2}0{18}}} +_{{}} {\text{DTM}}_{{{2}0{2}0}} - {\text{ DTM}}_{{{2}0{19}}} +_{{}} {\text{DTM}}_{{{2}0{21}}} - {\text{ DTM}}_{{{2}0{2}0}} } \right)/{5}$$

The topographical wetness index was calculated with the SAGA GIS wetness algorithm (Conrad et al. 2015) based on the DTM of 2021. The distance from the beach entrance was calculated as the normalized distance between each raster pixel (0.5 × 0.5 m) to the main beach entrance with 1 being the pixel closest to the entrance.

At each of the 30 locations (from here on referred to as experimental blocks) 9 plots of 0.5 x 0.5 m were created, resulting in a total of 270 plots spread out over the upper beach. Within each block the plots were placed on bare sand with a similar slope and 1 m between plots. While the same layout was followed for most locations (see Fig 1b) for some blocks a different shape was required as the space between existing adult vegetation was limited. When deviating from the standard layout, we adhered to the same placement requirements: bare sand, similar slope, and 1 meter distance between plots. To ensure unhindered recreation in the area the plots and blocks were left unmarked, instead they were georeferenced by means of a Real-Time Kinematic Positioning System (RTK, manufacturer: Topcon, 2 cm accuracy). Within each block the assignment of the different treatments to the plots was randomised. There were 8 vegetation treatments and one control in each block. The control was included to be able to correct for natural establishment within each block. The treatments consisted of 5 different species which occur naturally in the area. They were introduced via seeds and/or rhizomes based on their natural propagation method. For the three dune-building grasses *Ammophila* *arenaria,* *Elytrigia* *juncea,* and *Leymus* *arenarius,* both seeds and rhizomes were introduced as separate treatments, whereas *Honckenya* *peploides* and *Cakile* *maritima* were introduced only by means of seeds (Table 1, Fig 1d and e)*.* Accidentally, one out of the 30 blocks did not have a *Leymus* seeds treatment. As a result, this block has been excluded from all analyses conducted at block level. The study species were chosen to represent most common plant species on the beach, forming a cross section of beach plant diversity. The species are a mix of grasses and herbaceous species, and they comprise both of pioneer (*Elytrigia* and *Cakile*) and early successional species (*Ammophila,* *Leymus,* and *Honckenya*) common to beaches across Europe. Additionally, they vary in growth form, lifespan and embryo dune shaping abilities (Greipsson and Davy 1994, 1995; Gagné and Houle 2002; Davy et al. 2006; van Puijenbroek et al. 2017a, b; Nolet and Riksen 2019; Reijers et al. 2020; Ievinsh and Andersone-Ozola 2020; Huisman et al. 2021; Lammers et al. 2023).

**Table 1:** Overview species and introduced material in terms of the number of seed or rhizomes nodes per plot

Species scientific name(s)	Common name	Seed material (g/plot)	Shoot emergence potential	
			Seeds	Rhizome nodes
*Ammophila* *arenaria*, *Calamagrostis* *arenaria* *(L.)*	(European) Marram grass	5.00 ± 0.05	1055	40
*Elytrigia* *juncea*, *Elymus* *farctus* *boreoatlanticus* *(L.)* *Roth,* *Elymus* *junceiformis,* *Elytrigia* *junceiformi*	Sand-couch grass	9.00 ± 0.05	214	40
*Leymus* *arenarius*	Lyme grass	9.00 ± 0.05	645	30
*Honckenya* *peploides*	Sea sandwort	2.00 ± 0.05	175	–
*Cakile* *maritima*	European sea rocket	3.50 ± 0.05	137	–

### Plant material

Seeds of all species were manually collected at the Sand Engine in August 2021. They were stored at ambient outside temperatures and humidity until field introduction in early April 2022. Rhizomes of the dune-building grasses were collected maximally 2 days prior to the field introduction and stored at ambient soil temperatures. Each rhizome piece was cut to contain 2 nodes. Preprocessing of the seeds mimicked natural dispersal of the plant species. For *Ammophila* (Huiskes 1977) and *Leymus* seeds naturally fall out of the inflorescence, while for *Elytrigia* *juncea* the seeds remain in their husks and break off from the stem inflorescence (Homberger et al. 2025). Therefore, the inflorescences of *Ammophila* and *Leymus* seeds were threshed mechanically and then manually sieved to obtain the seeds. In contrast, the inflorescences of *Elytrigia* *juncea* were broken into pieces, and the seeds within the husks were manually separated from the stem parts. *Cakile* seed pods were removed from the stems as they normally seem to break off the plant (Davy et al. 2006). Finally, the *Honckenya* seeds were left untreated.

The *Ammophila* and the *Elytrigia* rhizome treatment plots each contained 20 rhizome pieces (40 nodes). The plots with *Leymus* rhizomes contained only 15 rhizome pieces (30 nodes), due to their larger size and the dimensions of the plots. The number of seeds per individual plot was calculated based on the average number of seeds per gram of material after pre-treatment. For the amounts of seeds or rhizomes introduced per plot see Table 1. We assumed that each seed or rhizome node has the potential to emerge as a shoot.

### Vegetation and environmental measurements

During June (May 31–June 3), August (16–17) and October (3–6) 2022, the shoot numbers, bed level, and soil moisture were assessed. During each monitoring round, the emerged shoots in each plot were counted. Shoot counts from control plots were subtracted from treatment plots to correct for spontaneous establishment from non-introduced diaspores. If the control had more shoots than the treatments, the corrected plant number was set to zero. At this early life stage, it was not possible to identify the grasses at species level without significantly damaging them. Therefore, all grass treatments were corrected for the total amount of grass shoots found in the control plot. *Honckenya* *and* *Cakile* were clearly identifiable. Their numbers were corrected directly with the numbers found in the control plot. For data visualisation, the control-corrected shoot count was expressed as the percentage of the number of introduced diaspores, from here on referred to as plant success. Therefore, plant success (i.e., establishment) is based on the number of emerged shoots relative to the amount of introduced material (seeds or rhizomes) per plot. Values over 100% can be reached when rhizomes develop additional nodes with shoots across the growing season, exceeding the two nodes present at introduction (see 2.3, section plant material). The number of emerged species per block was used as a proxy for biodiversity. This was chosen over common biodiversity indices since those formal indices include the abundance of individual species. Given the difference in the number of introduced diaspores between the species in our experiment, we wanted to avoid a biased comparison.

To separate the impact of recreation pressure from key environmental drivers, the soil moisture and bed level change were measured at each monitoring moment for each plot. The soil moisture of the top 5 cm was measured with a W.E.T. sensor kit containing a HH2 moisture meter and a WET-2 sensor (manufacturer: DELTA-T Devices LTD, 3% accuracy). To determine the plot bed level, the NW and SE corner of each plot were measured by means of RTK and took the average of the two heights. Bed level change was determined as the difference between bed level during a monitoring moment and the initial bed level at the time of planting in April. Lastly, the distance to the sea relative to the high-water line was determined from satellite images using QGIS 3.28.8 (QGIS Association 2022), and ranged from 173.4 to 359.3 m. Embryo dune initiation was assessed visually as the difference of the height of the plot in contrast to its surroundings during the October measurement moment.

### Visitor counts and pathway determination

First the network of main pathways was visually assessed in the field. Then visitors were counted and it was noted whether or not they were on the identified main pathways. To ensure good visibility during visitor counting, the research area was divided into 11 sections with equal surface areas, marking the corners of each section with sticks as a visual reference. Within each section, visitors were counted 5 times for 15 min across the 29th and 30th of July 2022. The weather conditions during counting were favourable for recreation (dry and mostly sunny). Goossen et al. (2020)reports the highest number of visitors at the Schelpenpad entrance in the months July and August. To be able to calculate the distance of plots to the main pathways they were delineated based on footstep intensity visible on the imagery from an unmanned aerial vehicle (UAV). The RGB images were taken with a DJI Mavic pro 2 (manufacturer: DJI) on July 30th, 2022, and geo-referenced using the RTK. An orthomosaic was created with Agisoft Metashape Professional (2.2.0) (AgiSoft 2024), followed by the manual delineation of the pathways in QGIS. For the delineation the pathways were defined as interconnected areas with high numbers of footsteps. High numbers of footsteps were defined as areas where there were no individual footsteps visible but rather many overlapping ones (Fig 1c). To investigate the effects of visitor pressure on the establishment of plants we determined the ‘distance to the pathway’. This distance was defined as the shortest distance to the edge of the closest pathway for each plot, resulting in a path distance range of 0–72.3 m, where 8 of the 29 blocks were (partly) located on the pathways.

### Statistical data analyses

R (4.3.1) (R Core Team 2024) was used for data pre-processing, analyses, and visualisation. For the data organisation we used the R packages “openxlsx” (Schauberger et al. 2023) and “dyplr” (Package “Dplyr”: A Grammar of Data Manipulation 2023). Data visualisation was done with “ggplot2” (Wickham et al. 2024), “gratia” (Simpson 2024) and “ggpubr” (Kassambara 2023). Several different types of statistical data analyses were done. The testing of visitor use on and off-pathway, differences in plant success, embryo dune initiation, and species count, as well as differences in plant success between treatments was done by means of a Kruskal-Wallis with a pairwise Wilcoxon ranks sum test.

A general additive model (GAM) (“mgcv” (Wood 2023)) was used to analyse the relationships between distance to the main pathways and plant success, using the environmental drivers as covariates (for the model equation see Eq. 2 in the Online Appendix). Separate analyses were done for the different monitoring moments in June, August, and October to prevent issues with repeated measures. The GAM model predicts the average number of shoots in a plot. The variables used in the analyses were: treatment; path distance; soil moisture; bed level change; distance to the sea; and the interaction of treatment with path distance. Block was included as a random effect to account for spatial autocorrelations between plots in the same block. As the data is count data, a negative binominal distribution was selected (Zuur et al. 2009). As smoothness selection method “REML” (Wood 2010) was used, and model selection was done using the double penalty approach by (Marra and Wood 2011). This is a “shrinkage” method for automatic term selection. Model assumptions were checked using “ DHaRMa” (Hartig and Lohse 2022). The concurvity was checked at a level of 0.5 (Ramsay et al. 2003), based on this, elevation was removed from the model in favour of sea distance, as the later was a better predictor. Testing for homogeneity of variance showed no significant quantile deviations for the simulated residuals of individual predictors for the June and October models but did for August visual inspection show these to be minimal, see Figs 8, 9 and 10 in the Online Appendix. Other model assumptions showed there were no violations for outliers, remaining spatial autocorrelation or zero-inflation. There is significant under dispersion for the August (*p* < 0.016) and October (*p* < 0.0001), however, this should not cause issues for the model interpretation as it leads to more conservative estimates.

## Results

### Pathways

**Fig. 2.**
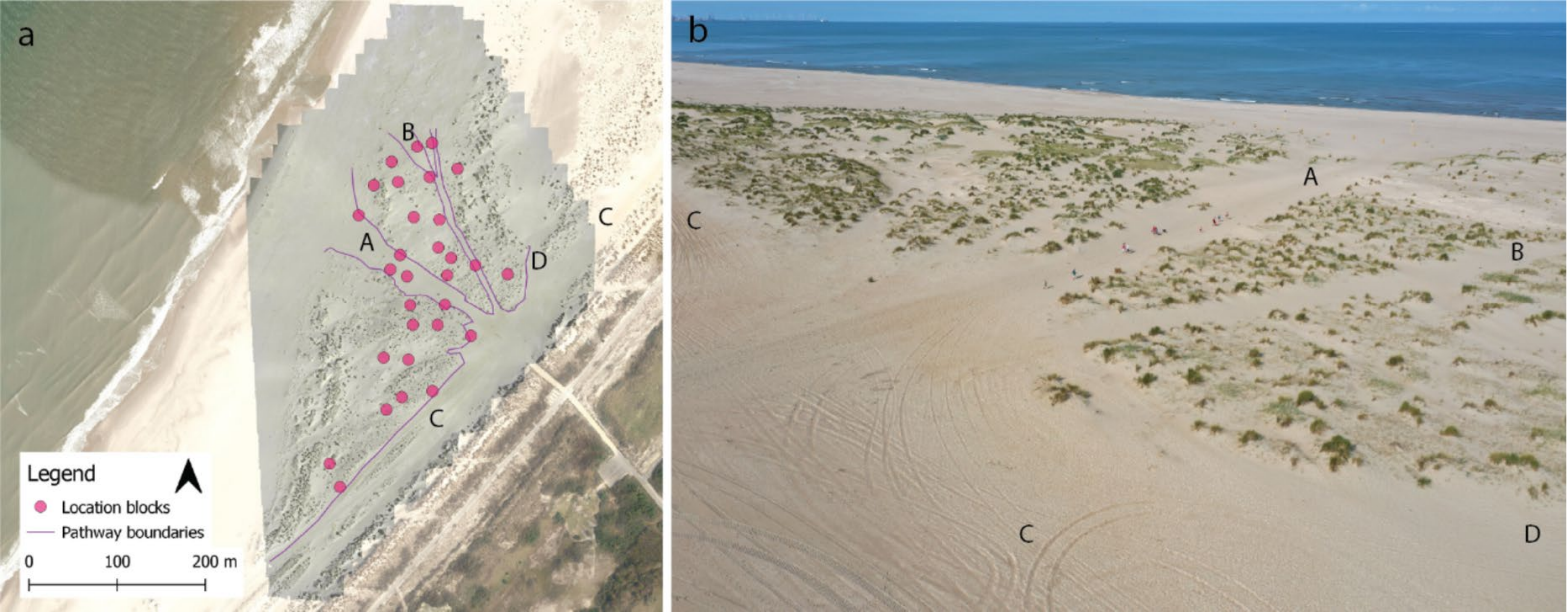
**a** Orthomosaic of research area at the Schelpenpad beach entrance (July 30th, 2022) with the different research blocks in pink and the four delineated main pathways. **b** Areal image of the research area with the identified pathways. The different identified pathways **A** pathway to the sea, **B** pathway along the seaward toe of the embryo dune row, **C**, pathway parallel to the foredune, **D** pathway towards the lake

We identified four main pathways in the study area (Fig 2). Of the two largest, one led directly from the beach entrance to the sea (A) and the other ran parallel to the foredune (C). The two smaller pathways followed the seaward toe of an embryo dune row (B) and led from the beach entrance towards the lake (D). The latter pathway is partly merged with the pathway parallel to the foredune in the study area beach section. During the visitor count, a total of 407 visitors were observed, 80.6% (328) of the beach visitors were counted on one of the four main pathways, confirming most recreation pressure is located on and near these pathways. The other 19.4% of visitors were counted in the areas in between the pathways. Moreover, the pathway leading directly to the sea (A) was the busiest, it was used by 46.2% (188) of the visitors. Observed visitor groups were: dominantly pedestrian adults and children, dogs, and occasional off-road vehicles, for a detailed group counts see Table 4 Online Appendix. Two vehicles were observed on pathway C and one on A during the counting. During general observations over the course of the fieldwork these were the dominant pathways used by vehicle traffic. Pathways B and D were also used on occasion. These observations match those made by (Goossen et al. 2020). Furthermore, we did not observe vehicles outside the pathway boundaries in the research area. Due to the low number of vehicles and their restricted motion patterns, we assume the found patters with regard to on and off the pathways are caused by the individual beach visitors trampling.

### General vegetation establishment

The plant success of individual plots ranged between 0 and 121.5 %, with an overall mean success of 3.8%. There were large differences in mean success between species and diaspore types (Table 2). The grasses performed better than the herbs *Cakile* and *Hockenya*, which showed the lowest success rates throughout the experiment. The highest success was found for *Elytrigia* seeds with a much higher success rate than any of the other treatments. Overall, a higher success of seeds versus rhizomes for the grass species can be observed (*Χ*^*2*^ = 104.16, *df* = 1, *p* < 0.001). At a species level this holds for both *Elytrigia* and *Leymus*. Only, *Ammophila* rhizomes have a higher success than the *Ammophila* seed treatment. Moreover, for all treatments there is a general dip in success rates in August, most likely related to the dry weather conditions in the weeks prior to the measurements (Huiskamp 2023) (Online Appendix Fig 6).

**Table 2:** Mean (standard deviation) of the plant success percentage at different measurement moments and for different treatments

Diaspore	Species	June	Augst	October	Species means	Group means
Rhizomes	*Ammophila* *arenaria*	1.5 (3.6)^bd^	1.3 (4.6)^b^	3.6 (15.5)^bd^	2.1 (9.5)^def^	1.9 (6.4)^a^
	*Elytrigia* *juncea*	1.3 (2.5)^bde^	0.8 (1.9)^bc^	1.3 (2.8)^bd^	1.1 (2.4)^defgh^	
	*Leymus* *arenarius*	4.7 (6.8)^cefg^	1.4 (3.2)^bc^	1.7 (3.4)^bd^	2.6 (4.9)^efgh^	
Seeds	*Ammophila* *arenaria*	1.7 (1.5)^cef^	0.9 (1.5)^bcd^	2.3 (2.5)^ce^	1.6 (2.0)^a^	7.8 (13.1)^b^
	*Elytrigia* *juncea*	15.0 (11.3)^a^	12.6 (13.7)^a^	23.0 (25.8)^a^	16.9 (18.4)^b^	
	*Leymus* *arenarius*	6.2 (6.9)^fg^	4.0 (5.2)^cd^	4.3 (5.7)^bde^	4.8 (6.0)^c^	
	*Cakile* *maritima*	0.4 (0.5)^bde^	0.3 (0.5)^bc^	0.4 (0.9)^bd^	0.3 (0.7)^defh^	0.6 (1.1)
	*Honckenya* *peploides*	1.3 (1.7)^cdef^	0.2 (0.5)^bc^	0.9 (1.3)^bde^	0.8 (1.3)^fgh^	
Mean		4.0 (7.1)	2.7 (6.8)	4.6 (13.0)	3.8 (9.4)	

The plant success is based on the number of emerged shoots relative to the amount of introduced material per plot. Significant difference are indicated with letters for June (*Χ*^*2*^ *=* 83.068*,* *df* *=* 7, *p* *<* 0.001), August (*Χ*^*2*^ *=* 49.536, *df* *=* 7, *p* *<* 0.001), October (*Χ*^*2*^ *=* 71.65, *df* *=* 7, *p* *<* 0.001), and species mean (*Χ*^*2*^ *=* 191.98, *df* = 7, *p* *<* 0.001) based on Kruskal-Wallis

### Plant success and recreation

**Fig. 3:**
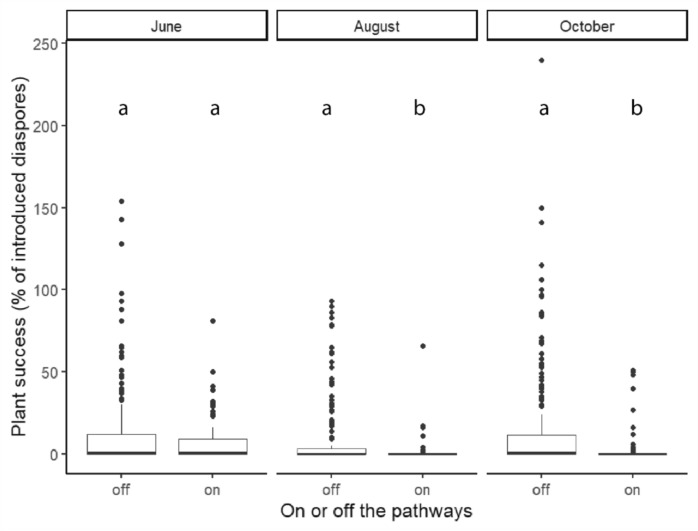
Plant success on and off the pathways per monitoring moment. The plant success is based on the number of emerged shoots relative to the amount of introduced material per plot, therefore it is possible to have multiple shoots from the same rhizome piece or seed rather than just one and thus a higher than 100% success. The middle line indicates the median and the box the 25th −75th quantile, the letters indicate the significant differences between groups for each moment.

We evaluated the effect of recreation pressure on plant success by comparing plant success on and off pathways and in relation to distance to the pathways. In early June, two months after the start of the experiment, the plant success in plots on pathways did not differ significantly from plant success off pathways (*Χ*^*2*^ = 1.74, *df* = 2, *p* = 0.19). By August and in October however, plant success on the pathways became significantly lower than off the pathways (August: *Χ*^*2*^ = 17.61, *df* = 2, *p* < 0.001, October: *Χ*^*2*^ = 18.4, *df* = 2, *p* < 0.001) (Fig 3). Additionally, plant success fluctuated over time. For on-pathway plants the success gradually decreased, while off-pathway the plant success declined from May to August but increased again in October as new shoots emerged from seeds and rhizomes.

**Fig. 4:**
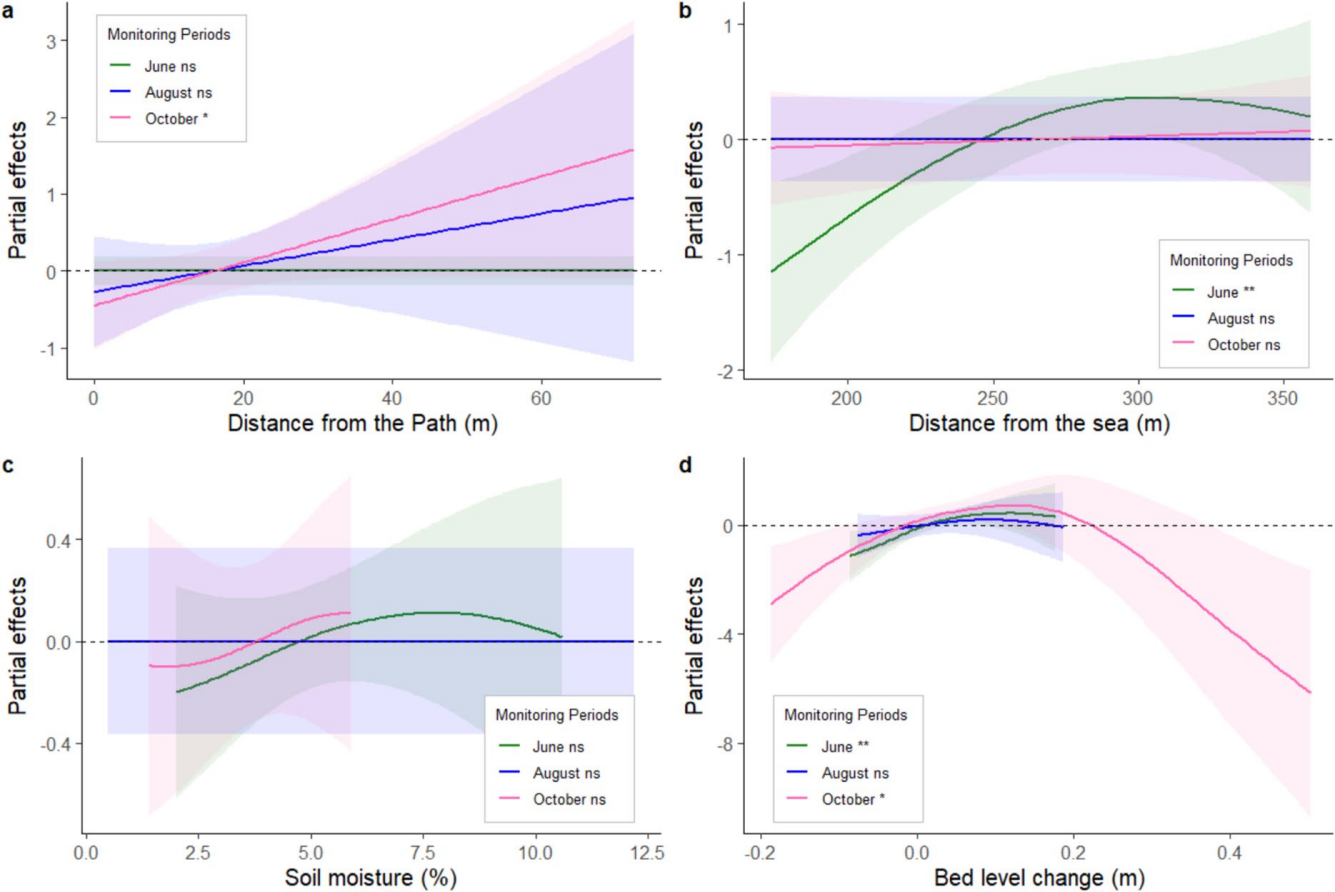
Modelled relationships of shoot number response using GAM models by means of the partial effects of different smoothers relative to the mean effect per monitoring moment, **a** distance to the main pathways, **b** distance to the sea, **c** soil moisture, and **d** bed level change. Above the horizontal dotted line at the 0 indicates a positive effect, below a negative effect of the variable. The shaded areas indicate the 95% confidence interval.

The GAMs for different measurement moments (Table 3, for detailed overview model see Online Appendix Tables 5, 6 and 7) showed significant effects of recreation pressure, distance to the sea, and bed level change on the number of shoots. However, there were no significant effects of soil moisture. For all monitoring moments, treatment was a main explanatory factor for the number of shoots (Table 3). The effect of recreation pressure, as expressed in distance to the pathway, was significant in October. There was a negative impact of the pathways up to a 20-meter distance, as can be seen from the path distance smoother in Fig 4a. There were no significant additive interaction effects between specific treatments and path distance at any of the monitoring moments. The absence of interactions effects suggests that the effect of path distance does not differ between treatments and followed a pattern similar to that of the main effect of path distance. Another driver important for the success rate of the plants was the distance to the sea. In June, a positive effect of increasing distance from the sea on shoot numbers was found (Fig 4b). Though it did not contribute significantly to plant success in future sampling periods. Bed level change is the third driver which was a significant contributor to the success of plant establishment. Across the growing season bed level changes ranged between -0.19 and 0.5 m. A significant impact could be found in June and October. At both moments the models predict a positive effect at low amounts of burial, in October this is up to ± 20 cm of burial. Erosion had a negative effect on shoot numbers in June. In October, only very low amounts of erosion (±2 cm) showed a positive effect while higher levels of erosion were disadvantageous to the shoots (Fig 4d). Soil moisture, which ranged from 0.5 to 12.2 %, was not significant in any of the models (Fig4c) (data plots see Online Appendix Figs. 11, 12, 13 and 14).

**Table 3:** Summary of the results of the smooth terms of the generalized additive models (GAMs) predicting the (interactive) effects of multiple variables on shoot counts for each monitoring moment

		Monitoring moment		
		June	August	October
Smoother terms				
Main drivers	Path distance	ns	ns	*
	Bed level change	**	ns	*
	Sea distance	**	ns	ns
	Soil moisture	ns	ns	ns
Interactions path distance with treatments	*Ammophila* *arenaria* rhizomes	ns	ns	ns
	*Ammophila* *arenaria* seeds	ns	ns	ns
	*Elytrigia* *juncea* rhizomes	ns	ns	ns
	*Elytrigia* *juncea* seeds	ns	ns	ns
	*Leymus* *arenarius* rhizomes	ns	ns	ns
	*Leymus* *arenarius* seeds	ns	ns	ns
	*Cakile* *maritima* seeds	ns	ns	ns
	*Honckenya* *peploides* seeds	ns	ns	ns
Parametric terms				
Treatments	*Ammophila* *arenaria* rhizomes	*	***	**
	*Ammophila* *arenaria* seeds	***	***	***
	*Elytrigia* *juncea* rhizomes	ns	ns	ns
	*Elytrigia* *juncea* seeds	***	***	***
	*Leymus* *arenarius* rhizomes	*	ns	ns
	*Leymus* *arenarius* seeds	***	***	***
	*Cakile* *maritima* seeds	ns	ns	ns
	*Honckenya* *peploides* seeds	***	ns	**
Random factors	Block	***	***	***
Deviance explained		74.4%	83.2%	78.3%

Significance codes: *** < 0.001; ** < 0.01; * < 0.05; ns = not significant

### Spatial patterns in establishment

**Fig. 5:**
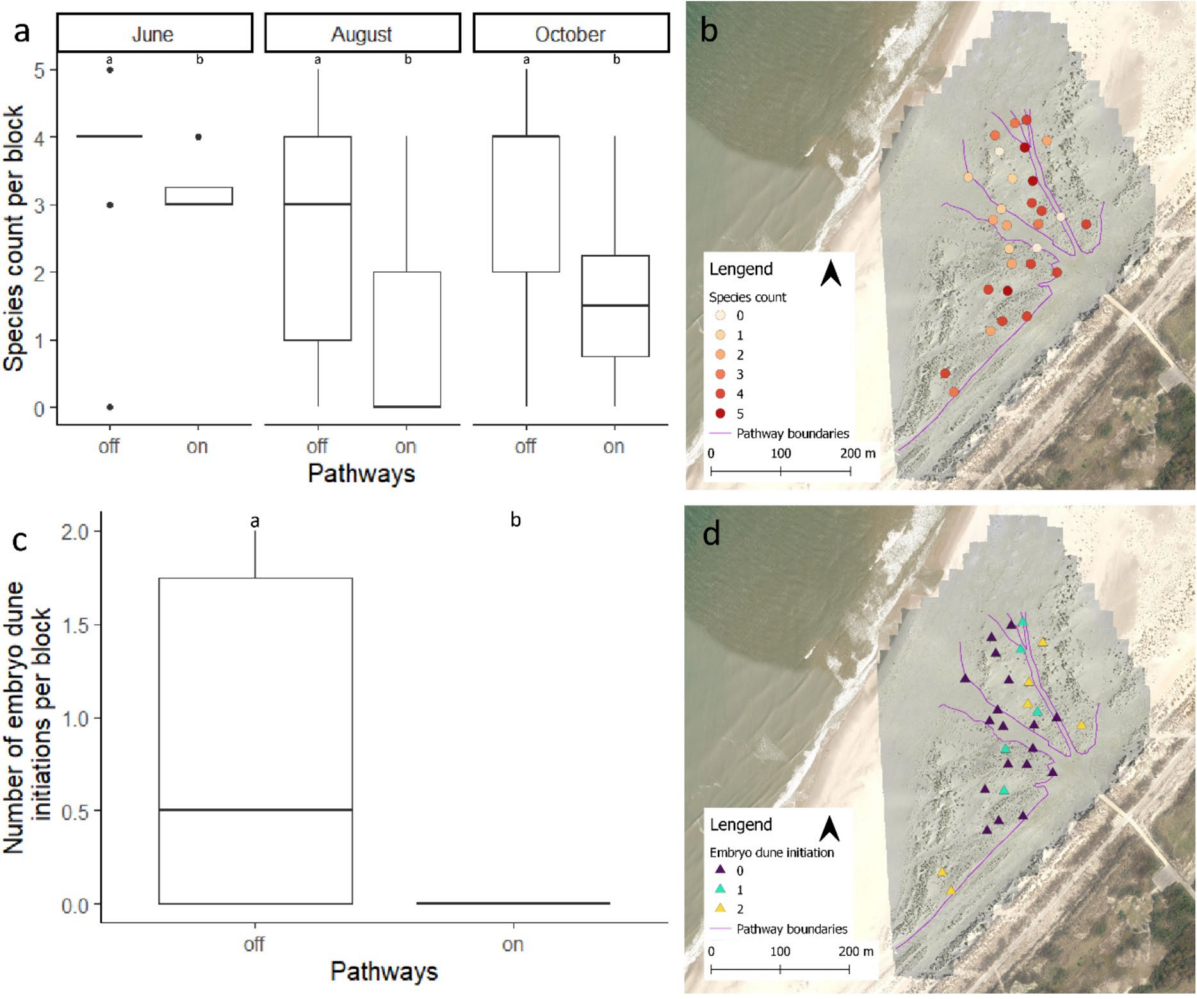
**a** On and off pathway differences in species counts, expressed as emerged number of species from seeds, per monitoring moments. The middle line indicates the median and the box the 25th−75th quantile, the letters indicate the significant differences between groups for each moment. **b** Spatial distribution of species counts expressed as emerged number of species from seeds per block in October. **c** On and off pathway differences in the number of embryo dune initiations per block. **d** Spatial distribution of the embryo dune initiations per block in October.

Aggregating the results into the number of species established per block, the impact of the pathways becomes more pronounced (Fig 5a). We found a significantly lower number of species per block on (mean = 1.6 species/block) than off (mean = 3.2 species/block) the pathways for all monitoring moments (June *Χ*^*2*^ = 6.6089, *df* = 1, *p* = 0.01015, August *Χ*^*2*^ = 6.4802, *df* = 1, *p* = 0.01091, October *Χ*^*2*^ = 6.0332, *df* = 1, *p* = 0.01404). Moreover, the species number was lowest around the busiest pathway A (Fig 2), which leads directly to the sea (Fig 5b).

By October, there was embryo dune initiation in 37.9% of the 29 blocks. The initiation occurred in plots of two different treatments, *Elytrigia* (7) and *Leymus* seed (9), with a single exception of a plot with *Ammophila* rhizomes. There were no cases of embryo dune initiation on the pathways (*Χ*^*2*^ = 5.8, *df* = 1, *p* = 0.01603) (Fig 5b) the mean distance of embryo dune initiation was at 17.36 m distance from the pathway. The closest of embryo dune initiation was at 0.98 m from the edge, though this was at the edge of pathway B, which is much less frequented by visitors. The spatial patterns further showed that the locations of starting embryo dune formation coincided with the locations with the establishment of a high number of plant species.

## Discussion

We investigated the impact of anthropogenic recreational pressure through trampling by beach visitors on establishment and dune building potential of five common plant species of sandy coastal beaches by introducing seeds and rhizomes at different distances from existing, spontaneously developed, pathways. We found that the establishment of all species was significantly reduced up to 20 m from these pathways. Likewise, development of embryonic dunes in the plots occurred mostly away from the pathways. This area of anthropogenic influence is much smaller than the 120 m observed for beaches without pathways (van Rosmalen et al. 2025), suggesting that co-existence of recreation and nature values can be partly directed by guiding visitor flows across the beach.

### Anthropogenic pressure, pathways, and vegetation establishment

Our observations indicate that most beach visitors move from the beach entrance to the sea using the same route, thus creating and maintaining pathways. That pathways constrained anthropogenic pressure was also reflected in the establishment success of the vegetation. We found that at approximately 20 m off the pathways, the negative effects of anthropogenic pressure are no longer present. Furthermore, this effect became more negative over time, aligning with the onset of the beach season at the site, and confirming the role of recreation in explaining the observed spatial patterns of lower vegetation establishment near pathways. The zone of influence of 20, observed in our study is much less than the 120 m reported for sparsely vegetated beaches (van Rosmalen et al. 2025). At the sparsely vegetated beach there was no discernible pathway development. Rather the walking patterns were more diffuse and reflected a funnel-shape surrounding the most direct route from beach entrance to the sea (van Rosmalen et al. 2025). Together these findings suggest a positive feedback between recreation pressure, and vegetation establishment and dune building on the beach in our study area. Starting from an open beach, most visitors move from the entrance to the sea, creating variation in trampling pressure and thus variation in establishment opportunities for vegetation and development of topography. If vegetation develops on the beach, the dominant routes become visually reinforced, and pathways develop, further constraining visitors to these routes, reducing pressure outside the pathways. Analyses of spatial structure and development of tracks and pathways through time would enable further testing of this hypothesis.

We found no differences in the response of species and diaspores (seeds, rhizomes) to trampling. We had expected differences in their response to anthropogenic pressure, in line with their position in beach succession, with pioneer species being more resistant to stress, including trampling, than species later in the succession. Instead, our results suggest species are equally vulnerable to trampling as it affects establishment. It is possible that at different life stages these species are not equally susceptible to trampling as the initial establishment is an especially vulnerable stage of vegetation development. Long term monitoring at a larger scale could provide further insights into the continued development under recreation pressure at later life stages of the perennial species. In case of the initial establishment it implies that, in the absence of other strong environmental gradients, lowering anthropogenic pressure would benefit species establishment success in a similar manner. Thereby, a reduction of anthropogenic pressure will not only allow for vegetation establishment, but also increase biodiversity in the area.

Besides anthropogenic pressure other environmental drivers, notably sediment dynamics (change in bed level) contributed to the establishment of species in the study area. Both higher levels of burial and erosion negatively impacted the establishment success of the plant species which is in line with previous research (Homberger et al. 2025; van Rosmalen et al. 2025). Additionally, we found a positive effect of distance to the sea in early June. As the beach elevation in the research area is too high for inundation (4.5 m) this could point towards a general larger exposure, for example in temperature, closer to the sea, rather than a direct result from wave action.

### Mitigating anthropogenic pressure on landscapes

The number of species that successfully established away from pathways (mean = 3.2 species/block) was twice as high as the number that established on pathways (mean = 1.6 species/block). This suggests that trampling not only affects the total number of plant individuals but likely also affects biodiversity. These results echo those by research performed in inland dunes, who report a lower number of species and lower vegetation cover on pathways than off pathways across the world (Hylgaard 1980; Andersen 1995; Kutiel et al. 1999). In our study the impact on the vegetation was most pronounced on and around the main pathway leading from the foredunes to the sea. This suggests that anthropogenic pressure is not necessarily detrimental to plant diversity at low levels. However, at higher levels of recreation pressure the trade-off between biodiversity is more pronounced and the inclusion of pathways in an area can be a possible mitigation measure.

The spatial pattern of starting embryo dune formation echoed that of species establishment; dune initiation did not occur on the pathways and was predominantly absent from the busiest area. Nonetheless, our observations indicate that pathways can also facilitate the initiation of embryo dune formation and therefore enable topographical development. This makes sense as embryo dune formation is highly dependent on both sediment supply and sufficient plant cover (or number of shoots) (Hesp et al. 2019; Homberger et al. 2025). In our experiment, plots with embryo dune initiation have a mean cover of 363 shoots/m^2^, while plots without embryo dune initiation have a mean cover of 32 shoots/m^2^. Given the negative relationship between anthropogenic pressure, vegetation establishment success, and shoot numbers (van Rosmalen et al. 2025), it can be expected that with increasing recreation pressure a lower number of embryo dune initiations can be found. Dune initiation occurred almost exclusively in plots with *Leymus* and *Elytrigia* seeds. These two treatments combine both a high number of introduced diaspores with a high establishment success rate. Given that *Leymus* and *Elytrigia* are common pioneer dune-building species, our observations are relevant for understanding, and ultimately, predicting patterns in dune initiation along the North-Western European coastline (Greipsson and Davy 1994; van Puijenbroek et al. 2017a, b; Reijers et al. 2020)

In general, the establishment success in our field experiment is low, lower than reported shoot emergence in laboratory studies (Huiskes 1979; Greipsson and Davy 1995; Lim 2011; Del Vecchio et al. 2020; Bonte et al. 2021). A possible explanation can be the difference in field versus laboratory conditions as the establishment success is of a similar range to other field studies in the Netherlands (Homberger et al. 2025; van Rosmalen et al. 2025). The harsher environmental conditions in the field can cause lower germination rates can due to for example the large range in burial and erosion (− 19 to +50 cm) or the low soil moisture (0.5–12.2%) (Huiskes 1977; Lammers et al. 2024; Homberger et al. 2025). The low germination rates of *Cakile*, might additionally be affected by the lack of pretreatment the seed pods as they do not germinate well from intact pods Davy et al. (2006). Irrespective of the low general establishment success and environmental and anthropogenic stressors, the establishment success showed large differences between species and diaspores. Whereas *Leymus,* and especially, *Elytrigia* seeds are quite successful in establishing across the area, *Ammophila* seeds and the herb species *Cakile* and *Honckenya* did not do very well in our experiment. The contrast between the different grass seed treatments might lay in the viability of the seeds*.* The seeds of *Leymus* and *Elytrgia* are much larger in size and weight than those of *Ammophila*. Under erosive conditions, which result in the exposure of the seeds, heavier seeds are less likely to be caried away by aeolian (i.e., wind) transport. Furthermore, seeds of larger sizes are known to be more successful in germinating and establishing as they have a larger energy and nutrient reserve to draw from (Greipsson and Davy 1995, 1996; Moles and Westoby 2004). For rhizomes, a similar observation may apply as the success rate was highest, albeit not statistically significant, for species with the largest rhizome size: in order of success and size this is *Leymus,* *Ammophila,* and as smallest *Elytrigia*. Within the grass species, there were differences in which diaspore performed best. *Ammophila* and *Leymus* rhizomes outperform their seeds, while the seeds of *Elytrigia* outperform their rhizome counter parts, which is in line with previous research (Harris and Davy 1986a, b; Van Der Putten 1990). It is possible that this variance points towards a difference in resource allocation to the seeds and rhizomes and possibly a difference in reproductive strategy. Davy et al. (2006)

### Management and embryo dune resilience

Currently, the biodiversity and resilience of sandy coasts are under threat by multiple stressors. Under climate change, several of these stressors are expected to increase. Climate change is predicted to impact the vegetation and embryo dune establishment and growth through increased storm activities (Homberger et al. 2025) and through an increase in visitor numbers (Coombes and Jones 2010), both of these increases can lead to more frequent disturbances of the upper beach and embryo dune system, thus lowering the system’s capacity to maintain to sustain itself. Furthermore, urban areas behind the dune system in combination with the predicted sea level rise can lead to coastal squeeze, meaning the size of the accommodation space for plant growth on the upper beach will be reduced over time (Lansu et al. 2024; Nolet et al. 2018; Nolet and Riksen 2019). Management strategies should therefore focus on where upper beach vegetation and recreational space are desired and redirect visitor movements accordingly. If the presence of vegetation is undesirable, for example when foredune reinforcement is required, then directing people across this area will aid in keeping it open from vegetation. Thus, allowing the sediment to reach the foredune area more easily (Delgado-Fernandez 2011). In contrast, if vegetation is desired, for example for nature development and the physical requirements are met, then directing people away could mitigate the negative effects of visitors and enhance embryo dune development and biodiversity.

Our results imply that using pathways to mitigate the impact of recreation on vegetation biodiversity is possible on the upper beach. The pathways at our research area are delineated by adult vegetation and associated elevated topography. This suggests that pathways can be self-sustainable once formed. However, several matters should be noted with regard to the pathways on the Sand Engine:

- These pathways have formed organically in the area, suggesting that they direct visitors to places of interest. Therefore, when designing pathways across an area preference regarding why and how visitors move through areas should be considered to prevent the formation of unwanted ‘desire pathways’ (shortcut trails) (Ma et al. 2024; Bossowski et al. 2025).
- In the research area the pathways facilitating new establishment are delineated by adult vegetation. On a bare beach, initial vegetation establishment will require a different method to protect them from trampling. A study in Italy shows the usage of boardwalks for this purpose (Prisco et al. 2021). However, the tidal differences and storm surges in the Adriatic Sea are much smaller (Medvedev et al. 2020). Therefore, boardwalks might not be a sustainable method on low-laying beaches, such as in the Netherlands, where the tidal differences and storm surges are considerably larger (Jänicke et al. 2021). A larger tidal difference and higher storm surges would impact structures and the vegetation with a greater frequency and impact, increasing the coast/benefit ratio of using boardwalks. Possible alternatives with lower costs and maintenance requirements could be fences with wire, rushes, or even planted adult vegetation.
- The beach of the Sand Engine is unusually wide and high due to its artificial nature. It is possible that the width of the area effects how people move across it and that other drivers play a more significant role at smaller, low-laying beaches (Homberger et al. 2025; van Rosmalen et al. 2025). Replicating the experiment at beaches with more variable beach profile will further narrow down the scalability of our results.
- The number of visitors to the study area at the Sand Engine is smaller than the average on the Dutch coast due to its lower accessibility and lack of nearby facilities (Goossen et al. 2020). Therefore, it might be that at more intensely used areas soft pathway boarders are less or not sustainable to direct visitor movements, especially in combination with beaches with different beach profiles.

Finally two general limitations to this study, Firstly, a common ‘trampling’ factor, outside of the scope of this study, requiring further investigation is the impact of vehicles. Previous studies indicate that the impact of vehicles can be considerable and often larger than that of trampling by foot traffic (Anders and Leatherman 1987; Kutiel et al. 2000; Priskin 2003; Schlacher and Thompson 2008; Kindermann and Gormally 2010). Although, it is unclear how much the impact of vehicles versus foot traffic is both our observations as well as those by Bakhshianlamouki et al. (2024) suggests that the main activity by visitors is walking at the Schelpenpad area. As only a low number of vehicles compared to other beach visitors have been observed in the study area, therefore it can be assumed that majority of the found anthropogenic impact on the vegetation is the result of trampling by individual beach visitors rather than by vehicles. Secondly, the experiment covers a single tourism and growing season resulting in two considerations. Differences in yearly weather conditions can cause variability in the environmental conditions and visitor numbers, therefore also the strength of the negative effects from recreation on vegetation development is probably variable. Additionally, seasonal changes in beach usage are not accounted for within this study. Though, it should be noted that Goossen et al. (2020) found the summer to be the busiest part of the year in terms of recreation. This matches both the main growing season of the vegetation as well as the measurement period of this study. For management practices and policy to balance the landscape functions of nature development and recreation it is recommended to replicate the experiment at a larger temporal and spatial scale. Nonetheless, the positive effect of pathways on the initial vegetation establishment provides an important insight in how recreation pressure can be mitigated at the first stage of vegetation and topographical development.

## Conclusion

The use of pathways on the upper beach can help mitigate trade-offs between recreation and vegetation development in anthropogenic sandy coastal landscapes. This is achieved by localising most of the anthropogenic pressure to pathways, resulting in a reduction of the pressure by trampling in the wider area. In turn, this allows a higher number of plants and species to establish in the area, ensuing a higher biodiversity and greater chances for embryonic dune development. Thus, guiding visitor movement across the upper beach can contribute to the multifunctionality of beaches, enabling better coexistence of different landscape functions. The design of upper beach areas is therefore important for sustainable beach ecosystem and recreation management. The placement of facilities and pathways directs different functional usage of the upper beach by supporting or hindering vegetation and dune formation. To better understand interactions and landscape design choices in detail further research is required into the effect of the proposed management options at beaches with varying beach profiles, different amount of existing vegetation, and under different amounts and types of anthropogenic pressure.

## Supplementary Information

Below is the link to the electronic supplementary material.

Supplementary file1 (DOCX 850 KB)

## Data Availability

Data is available upon request.
