# Supplementary material for: Directing recreation pressure via pathways allows for coexistence of recreation and nature development on the upper beach
Source: Landsc Ecol. 2025 Nov 20;40(12):224. doi: 10.1007/s10980-025-02246-2 (PMC12634784; doi:10.1007/s10980-025-02246-2)
Supplement: Supplementary file 1 — Supplementary file1 (DOCX 850 KB) [file 10980_2025_2246_MOESM1_ESM.docx]

**Directing recreation pressure via pathways allows for coexistence of recreation and nature development on the upper beach**

**Appendix**

**Equation 2** Model equation

Model <- gam(PlantNumberCorrected ~ Treatment + s(BedLevelChange) + s(Moisture) + s(PathDistance) + s(SeaDistance) + s(PathDistance, by = Treatment) + s(BlockSA, bs = 're'), data = data, method = 'REML', select = TRUE, family = nb())


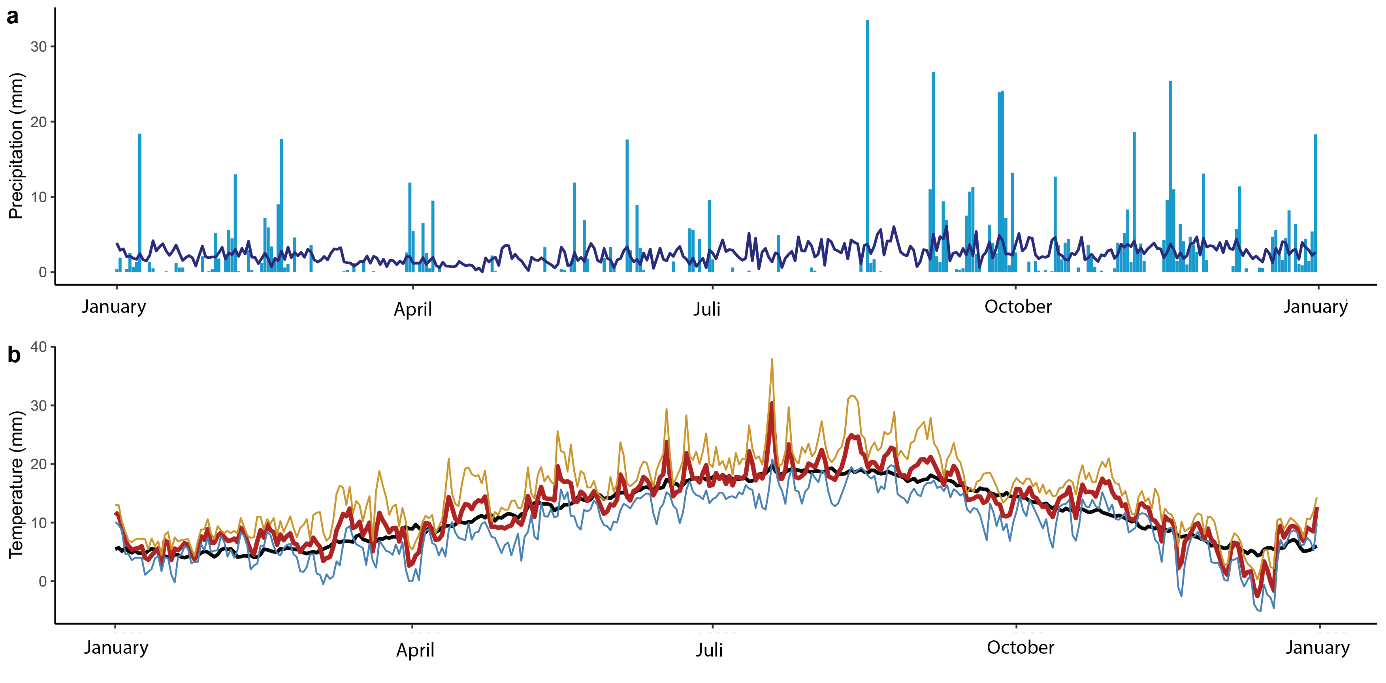


**Fig. 6** a) Daily precipitation in mm for 2022 (in light blue) and mean daily precipitation in mm in dark blue (2001-2024). b) Daily temperature in (°C), in red the mean, max in yellow, and min in light blue for 2022, and mean daily temperature in (°C) in black (2001-2024). Weather data during the experimental period retrieved from the KNMI (Royal Netherlands Meteorological Institute) station at Hoek van Holland (WMO code 06330 approximately 8 km away from the research area).


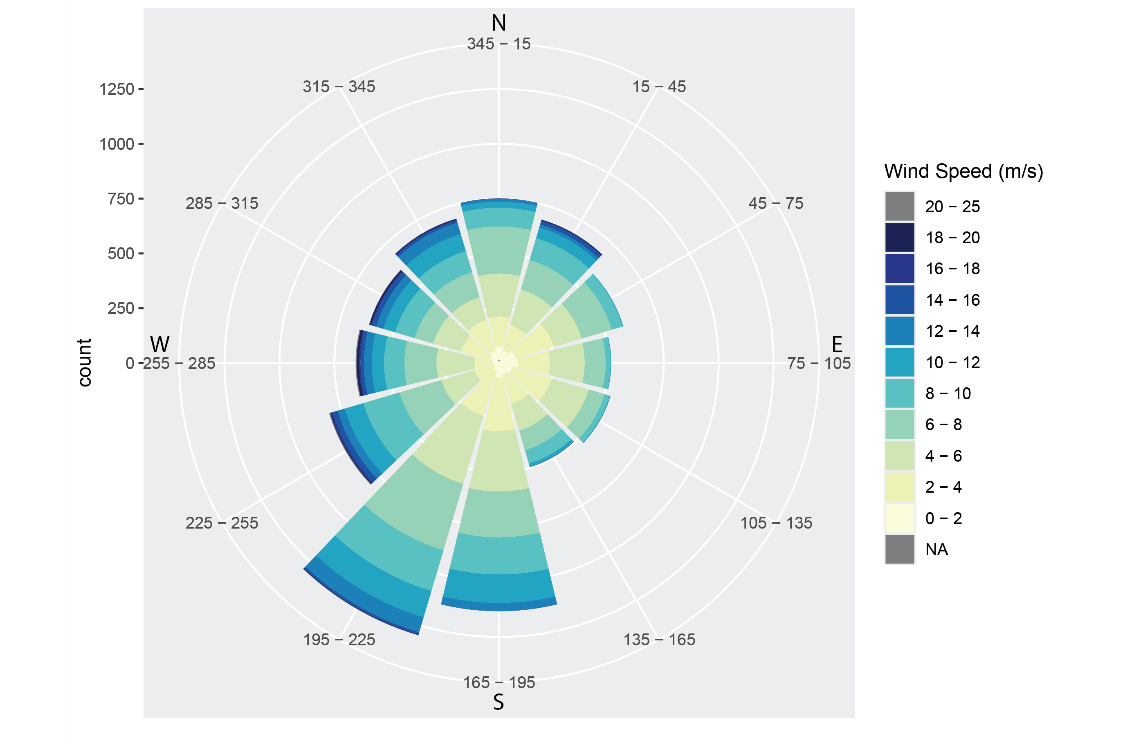


**Fig. 7** Wind speed and direction for 2022. Weather data during the experimental period retrieved from the KNMI (Royal Netherlands Meteorological Institute) station at Hoek van Holland (WMO code 06330 approximately 8 km away from the research area).

| 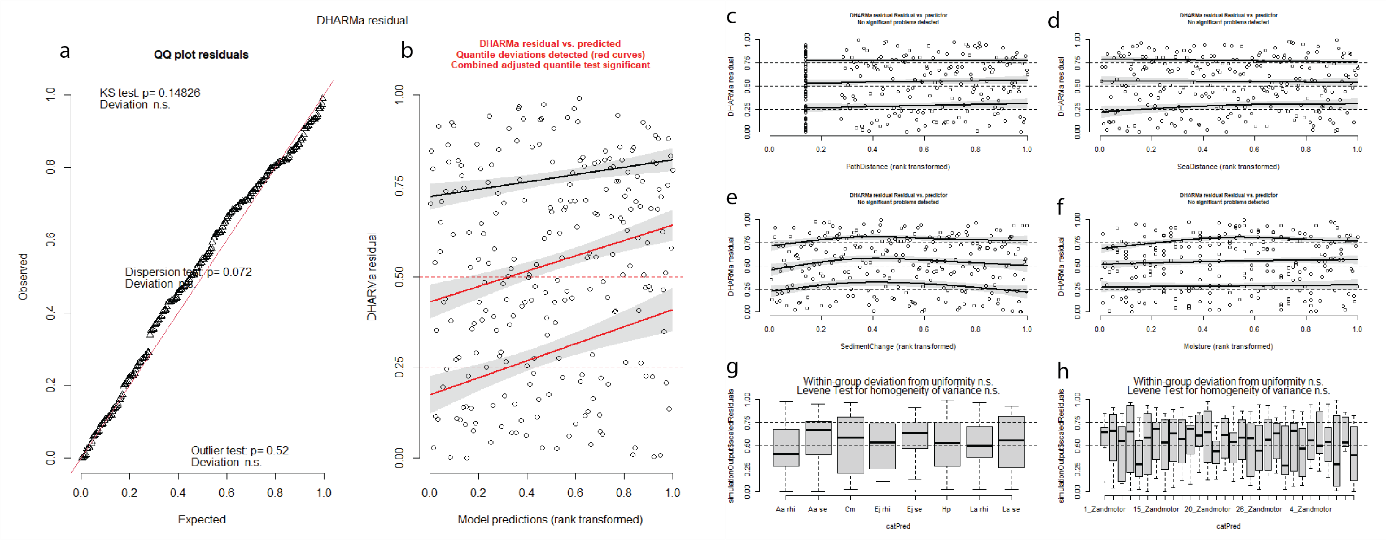 |
| --- |
| **Fig. 8** June model assumptions. a) K-S test, dispersion test, and outliers test. b-h) the simulated residuals vs model predictions with quantile deviations, the lines indicate the 25, 50 and 75 quantile, and the points the residuals vs the model prediction. For the whole model (b) and for individual model variable (c-h). |
| 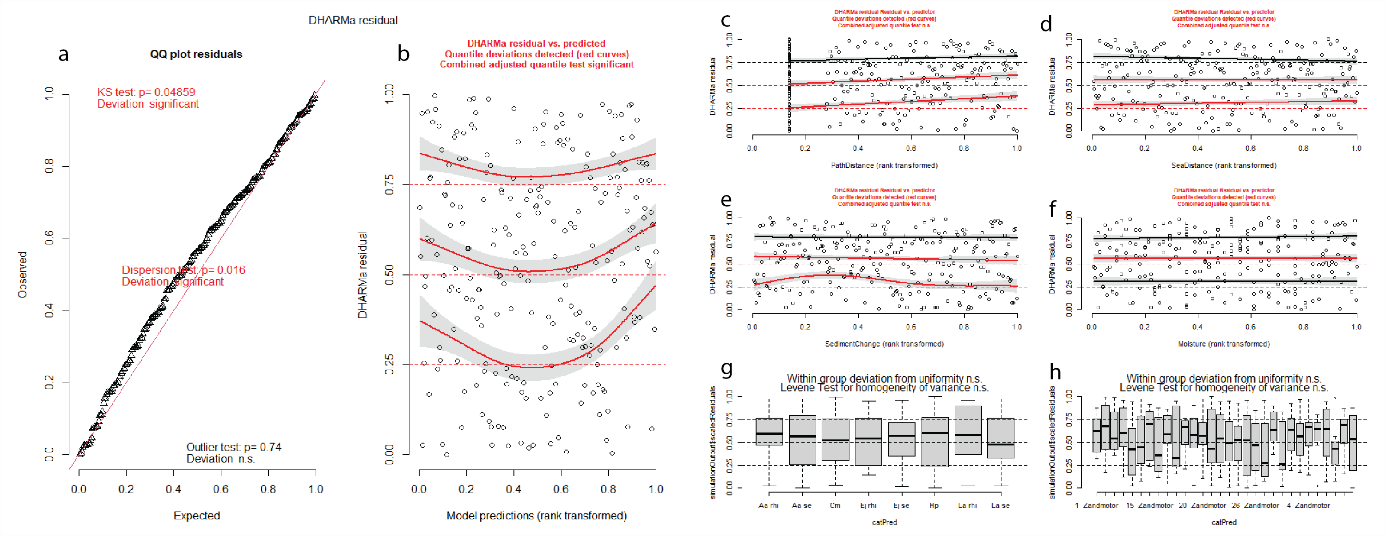 |
| **Fig. 9** August model assumptions. a) K-S test, dispersion test, and outliers test. b-h) the simulated residuals vs model predictions with quantile deviations, the lines indicate the 25, 50 and 75 quantile, and the points the residuals vs the model prediction. For the whole model (b) and for individual model variable (c-h). |

| 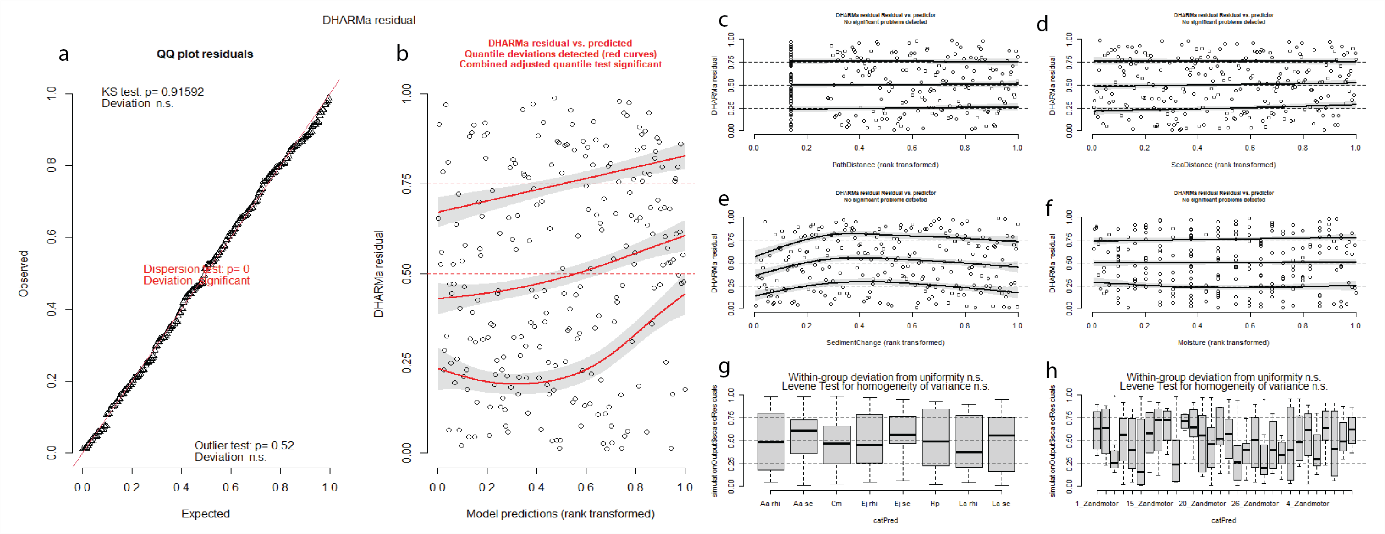 |
| --- |
| **Fig. 10** October model assumptions. a) K-S test, dispersion test, and outliers test. b-h) the simulated residuals vs model predictions with quantile deviations, the lines indicate the 25, 50 and 75 quantile, and the points the residuals vs the model prediction. For the whole model (b) and for individual model variable (c-h). |

**Table 4** Detailed visitor counts split into groups. (^a^ horseback rider)

|  | On road | Off road | Total |
| --- | --- | --- | --- |
| Stationary humans | 10 | 40 | 50 |
| Moving humans | 252 | 33 | 285 |
| Dogs | 62 | 6 | 68 |
| Vehicles | 3 | 0 | 3 |
| Other | 1^a^ | 0 | 1 |
| Total | 328 | 79 | 407 |

**Table 5** Model summary for plant number during the June monitoring moment

| Component | Term | Estimate | Std Error | z value | Pr(>\|z\|) |  |
| --- | --- | --- | --- | --- | --- | --- |
| A.Parametric coefficients: | (Intercept) | -0.84256 | 0.35654 | -2.363 | 0.01812 | * |
|  | Treatment Aa se | 348.124 | 0.38353 | 9.077 | < 2e-16 | *** |
|  | Treatment Cm | -0.02978 | 0.46292 | -0.064 | 0.94870 |  |
|  | Treatment Ej rhi | -0.01119 | 0.46497 | -0.024 | 0.98079 |  |
|  | Treatment Ej se | 412.889 | 0.38155 | 10.821 | < 2e-16 | *** |
|  | Treatment Hp | 138.376 | 0.40728 | 3.398 | 0.00068 | *** |
|  | Treatment La rhi | 0.84757 | 0.41933 | 2.021 | 0.04325 | * |
|  | Treatment La se | 408.266 | 0.38238 | 10.677 | < 2e-16 | *** |
| B.Smooth terms | **Term** | **edf** | **Ref df** | **Chi sq** | **p-value** |  |
|  | Bed level change | 1,83E+03 | 9 | 24.941 | 0.00400 | ** |
|  | Moisture | 7,73E+02 | 9 | 2.597 | 0.20585 |  |
|  | Path distance | 2,19E-02 | 9 | 0.000 | 0.85720 |  |
|  | Sea distance | 1,81E+03 | 9 | 54.700 | 0.00639 | ** |
|  | PathDistance:Treatment Aa rhi | 6,61E-01 | 9 | 0.000 | 0.55763 |  |
|  | PathDistance:Treatment Aa se | 3,80E-02 | 9 | 0.000 | 0.83081 |  |
|  | PathDistance:Treatment Cm | 2,35E-02 | 9 | 0.000 | 0.94475 |  |
|  | PathDistance:Treatment Ej rhi | 6,28E-02 | 9 | 0.000 | 0.51730 |  |
|  | PathDistance:Treatment Ej se | 7,19E+02 | 9 | 3.299 | 0.06624 | . |
|  | PathDistance:Treatment Hp | 7,61E-02 | 9 | 0.000 | 0.61435 |  |
|  | PathDistance:Treatment La rhi | 5,39E-02 | 9 | 0.000 | 0.82361 |  |
|  | PathDistance:Treatment La se | 2,94E-02 | 9 | 0.000 | 0.69936 |  |
|  | Block | 1,85E+04 | 29 | 52.584 | 3.2e-07 | *** |
| Signif. codes: 0 ‘***’ 0.001 ‘**’ 0.01 ‘*’ 0.05 ‘.’ 0.1 ‘ ’ 1 | | | | | | |
| R-sq.(adj) = 0.592 Deviance explained = 74.4% | | | | | | |
| -REML = 578.73 Scale est. = 1 n = 239 | | | | | | |

**Table 6** Model summary for plant number during the August monitoring moment

| Component | Term | Estimate | Std Error | z value | Pr(>\|z\|) |  |
| --- | --- | --- | --- | --- | --- | --- |
| A.Parametric coefficients: | (Intercept) | -29.993 | 0.8168 | -3.672 | 0.000241 | *** |
|  | Treatment Aa se | 36.104 | 0.7196 | 5.017 | 5.24e-07 | *** |
|  | Treatment Cm | 11.051 | 0.7645 | 1.446 | 0.148310 |  |
|  | Treatment Ej rhi | 0.2180 | 0.8253 | 0.264 | 0.791655 |  |
|  | Treatment Ej se | 50.946 | 0.7143 | 7.132 | 9.90e-13 | *** |
|  | Treatment Hp | 0.9016 | 0.7731 | 1.166 | 0.243481 |  |
|  | Treatment La rhi | 0.7920 | 0.7813 | 1.014 | 0.310741 |  |
|  | Treatment La se | 46.066 | 0.7148 | 6.445 | 1.16e-10 | *** |
| B.Smooth terms | **Term** | **edf** | **Ref df** | **Chi sq** | **p-value** |  |
|  | Bed level change | 8,43E+02 | 9 | 16.747 | 0.304 |  |
|  | Moisture | 4,12E-02 | 9 | 0.000 | 0.846 |  |
|  | Path distance | 4,85E+02 | 9 | 47.898 | 0.205 |  |
|  | Sea distance | 2,38E-02 | 9 | 0.000 | 0.922 |  |
|  | PathDistance:Treatment Aa rhi | 1,81E+03 | 9 | 5.472 | 0.170 |  |
|  | PathDistance:Treatment Aa se | 3,95E-02 | 9 | 0.000 | 0.829 |  |
|  | PathDistance:Treatment Cm | 2,72E-02 | 9 | 0.000 | 0.818 |  |
|  | PathDistance:Treatment Ej rhi | 6,57E+02 | 9 | 2.032 | 0.101 |  |
|  | PathDistance:Treatment Ej se | 4,20E-02 | 9 | 0.000 | 0.757 |  |
|  | PathDistance:Treatment Hp | 3,04E-02 | 9 | 0.000 | 0.639 |  |
|  | PathDistance:Treatment La rhi | 3,24E-02 | 9 | 0.000 | 0.811 |  |
|  | PathDistance:Treatment La se | 2,67E-02 | 9 | 0.000 | 0.679 |  |
|  | Block | 2,55E+04 | 29 | 169.079 | <2e-16 | *** |
| Signif. codes: 0 ‘***’ 0.001 ‘**’ 0.01 ‘*’ 0.05 ‘.’ 0.1 ‘ ’ 1 | | | | | | |
| R-sq.(adj) = 0.65 Deviance explained = 83.2% | | | | | | |
| -REML = 402.22 Scale est. = 1 n = 239 | | | | | | |

**Table 7** Model summary for plant number during the October monitoring moment

| Component | Term | Estimate | Std Error | z value | Pr(>\|z\|) |  |
| --- | --- | --- | --- | --- | --- | --- |
| A.Parametric coefficients: | (Intercept) | -19.534 | 0.5974 | -3.270 | 0.00108 | ** |
|  | Treatment Aa se | 43.014 | 0.5910 | 7.278 | 3.39e-13 | *** |
|  | Treatment Cm | 0.2980 | 0.6708 | 0.444 | 0.65681 |  |
|  | Treatment Ej rhi | 0.3274 | 0.6583 | 0.497 | 0.61895 |  |
|  | Treatment Ej se | 52.075 | 0.5861 | 8.885 | < 2e-16 | *** |
|  | Treatment Hp | 15.930 | 0.6143 | 2.593 | 0.00951 | ** |
|  | Treatment La rhi | 0.1351 | 0.6945 | 0.194 | 0.84579 |  |
|  | Treatment La se | 39.916 | 0.5944 | 6.715 | 1.88e-11 | *** |
| B.Smooth terms | **Term** | **edf** | **Ref df** | **Chi sq** | **p-value** |  |
|  | Bed level change | 2,97E+03 | 9 | 68.156 | 0.0106 | * |
|  | Moisture | 4,10E+02 | 9 | 1.030 | 0.2176 |  |
|  | Path distance | 7,87E+02 | 9 | 58.108 | 0.0325 | * |
|  | Sea distance | 1,61E+02 | 9 | 0.904 | 0.2874 |  |
|  | PathDistance:Treatment Aa rhi | 2,00E+03 | 9 | 6.237 | 0.0577 | . |
|  | PathDistance:Treatment Aa se | 4,39E-01 | 9 | 0.000 | 0.4766 |  |
|  | PathDistance:Treatment Cm | 1,25E+03 | 9 | 2.626 | 0.1456 |  |
|  | PathDistance:Treatment Ej rhi | 3,68E-02 | 9 | 0.000 | 0.9500 |  |
|  | PathDistance:Treatment Ej se | 4,02E-02 | 9 | 0.000 | 0.7973 |  |
|  | PathDistance:Treatment Hp | 4,19E-01 | 9 | 0.000 | 0.3583 |  |
|  | PathDistance:Treatment La rhi | 1,48E+03 | 9 | 2.667 | 0.1901 |  |
|  | PathDistance:Treatment La se | 4,68E-02 | 9 | 0.000 | 0.6881 |  |
|  | Block | 2,21E+04 | 29 | 96.431 | <2e-16 | *** |
| Signif. codes: 0 ‘***’ 0.001 ‘**’ 0.01 ‘*’ 0.05 ‘.’ 0.1 ‘ ’ 1 | | | | | | |
| R-sq.(adj) = 0.445 Deviance explained = 78.3% | | | | | | |
| -REML = 511.49 Scale est. = 1 n = 239 | | | | | | |


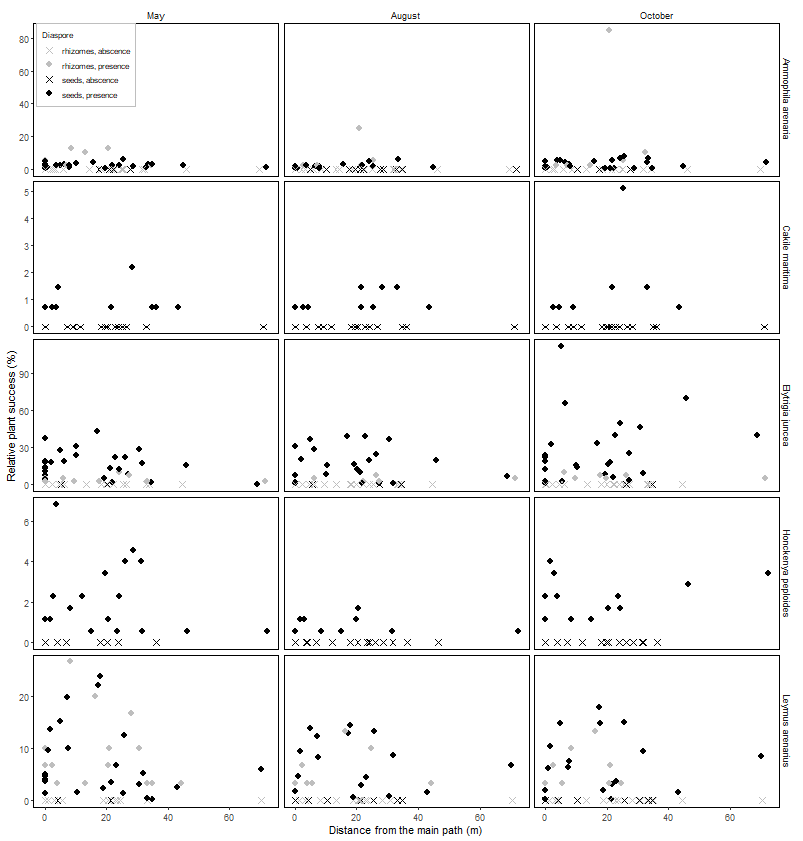


**Fig. 11** Relative plant success per plot (relative to the number of introduced diaspores) vs path distance per monitoring period for all treatments (diaspores x species), absence and presence of plants per plots are indicated for visual clarity in the lower success range


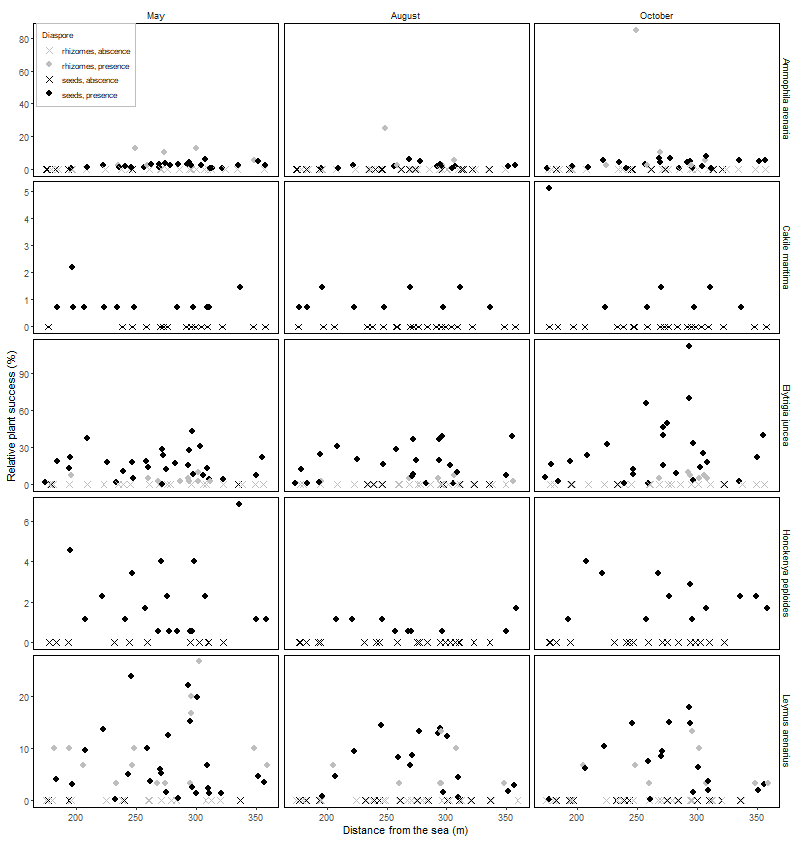


**Fig. 12** Relative plant success per plot (relative to the number of introduced diaspores) vs sea distance per monitoring period for all treatments (diaspores x species), absence and presence of plants per plots are indicated for visual clarity in the lower success range


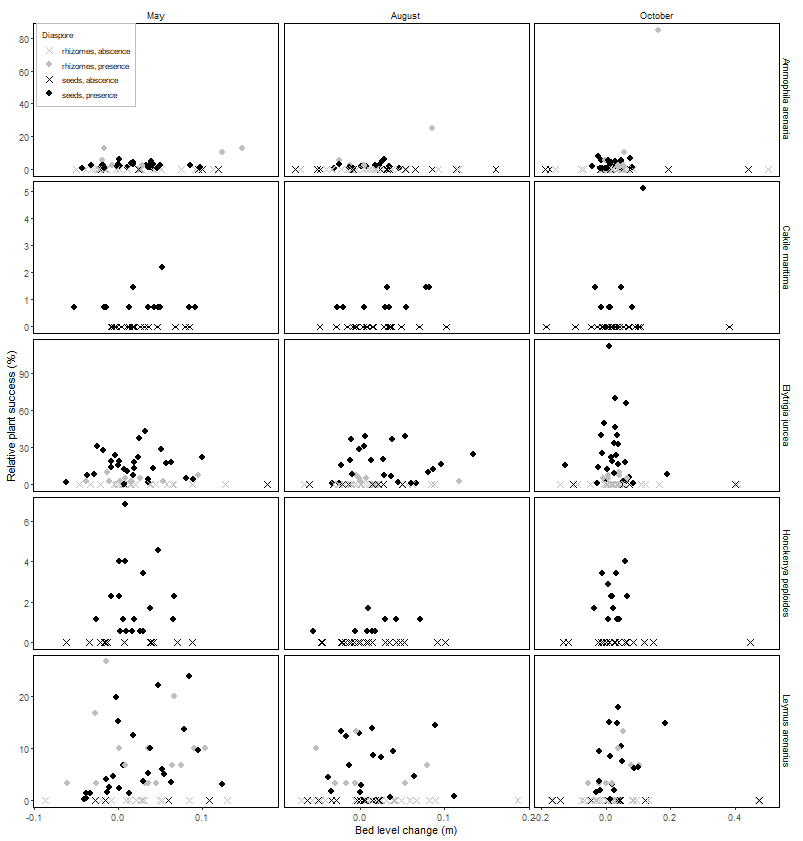


**Fig. 13** Relative plant success per plot (relative to the number of introduced diaspores) vs bed level change per monitoring period for all treatments (diaspores x species), absence and presence of plants per plots are indicated for visual clarity in the lower success range


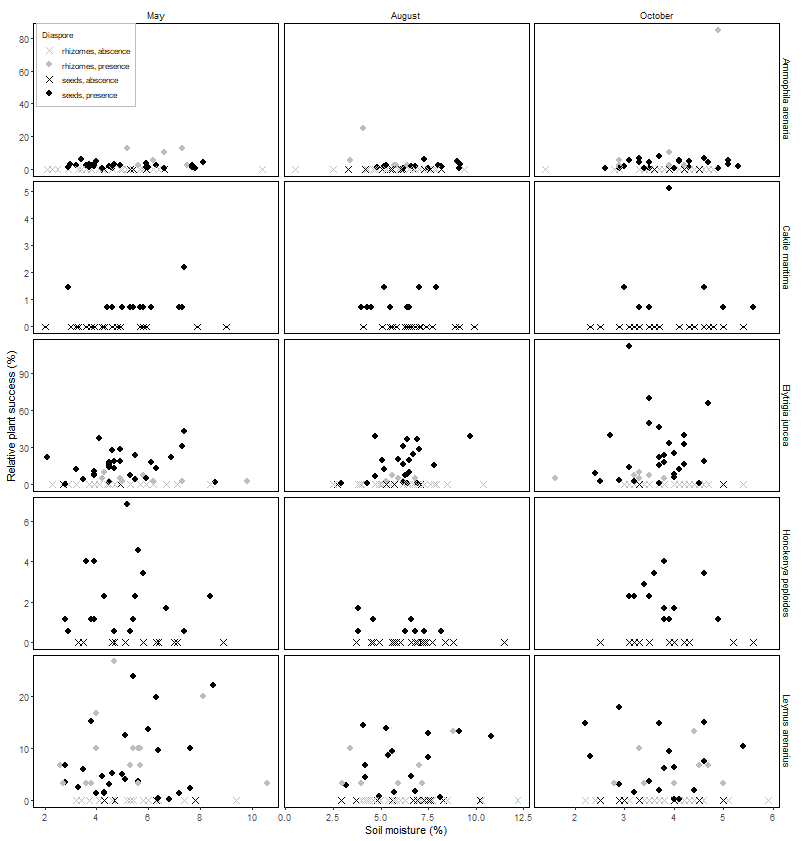


**Fig. 14** Relative plant success per plot (relative to the number of introduced diaspores) vs moisture per monitoring period for all treatments (diaspores x species), absence and presence of plants per plots are indicated for visual clarity in the lower success range
